# Impact of diabetes on three-year outcome after coronary stenting in patients with polyvascular atherosclerotic disease – a secondary analysis of the randomized TWENTE trials^☆^

**DOI:** 10.1016/j.ijcha.2025.101741

**Published:** 2025-07-06

**Authors:** Daphne van Vliet, Tineke H. Pinxterhuis, Eline H. Ploumen, Marlies M. Kok, Rosaly A. Buiten, Paolo Zocca, Ariel Roguin, Carl E. Schotborgh, Rutger L. Anthonio, Peter W. Danse, Edouard Benit, Adel Aminian, Carine J.M. Doggen, Clemens von Birgelen

**Affiliations:** a Department of Cardiology, Thoraxcentrum Twente, Medisch Spectrum Twente, Enschede, the Netherlands; b Department of Health Technology and Services Research, Faculty BMS, Technical Medical Centre, University of Twente, Enschede, the Netherlands; c Department of Cardiology, Hillel Yaffe Medical Center, Hadera and B. Rappaport-Faculty of Medicine, Israel, Institute of Technology, Haifa, Israel; d Department of Cardiology, Haga Hospital, The Hague, the Netherlands; e Department of Cardiology, Treant Zorggroep, Scheper Hospital, Emmen, the Netherlands; f Department of Cardiology, Rijnstate Hospital, Arnhem, the Netherlands; g Department of Cardiology, Jessa Hospital, Hasselt, Belgium; h Department of Cardiology, Centre Hospitalier Universitaire de Charleroi, Charleroi, Belgium; i Department of Cardiology, Ziekenhuisgroep Twente, Almelo and Hengelo, the Netherlands

**Keywords:** Coronary artery disease, Peripheral arterial disease, Polyvascular disease, Diabetes mellitus, Percutaneous coronary intervention

## Abstract

**Background:**

The presence of polyvascular atherosclerotic disease is associated with a high-risk of adverse events following percutaneous coronary intervention (PCI). As the extent to which the presence of diabetes further increases this elevated risk is unclear, based on current literature, we sought to assess the long-term outcome after PCI in patients with polyvascular disease, comparing those with and without diabetes.

**Methods:**

The current study population consists of patients with known polyvascular disease, identified from a pooled patient-level database of 4 PCI trials in all-comers treated with new-generation drug-eluting stents; no exclusion criteria were set. The main composite endpoint was major adverse cardiac event (MACE: any myocardial infarction, emergent coronary bypass surgery, clinically indicated target lesion revascularization, or all-cause mortality).

**Results:**

695 patients had polyvascular disease of whom 208(29.9 %) had diabetes. Patients with diabetes were older, had a higher body-mass-index, and had a higher prevalence of hypertension than those without diabetes. At 3-year follow-up, the incidence of MACE was significantly higher in polyvascular disease patients with diabetes (24.6 % vs.16.4 %, adj.HR:1.49, 95 %CI:1.05–2.12, p = 0.03), in particular insulin-treated patients, and was primarily attributable to a disparity in all-cause mortality which was more than twice as high in patients with diabetes (15.4 % vs.7.2 %, p < 0.001). Furthermore, the risk of repeated target vessel revascularization was higher in patients with diabetes (12.0 % vs7.0 %, adj.HR:1.88, 95 %CI:1.12–3.16, p = 0.02).

**Conclusions:**

In the high-risk population of PCI patients with polyvascular disease, the presence of diabetes represents a profoundly significant additional risk factor at long-term follow-up, associated with significantly higher adverse event risks.

**Trial registration:**ClinicalTrials.gov NCT01066650 NCT0133170 NCT01674803 NCT02508714.

## Highlights

• Diabetes rises event risk after coronary stenting in polyvascular disease patients.
• We examine to which extent diabetes increases the already elevated event risk.
• Diabetes rises the event risk in polyvascular disease patients by > 50 %.
• This is mainly attributable to more deaths from any cause.
• These data suggest that strong preventive measures are warranted in these patients.

## Graphical abstract

**Figure:**
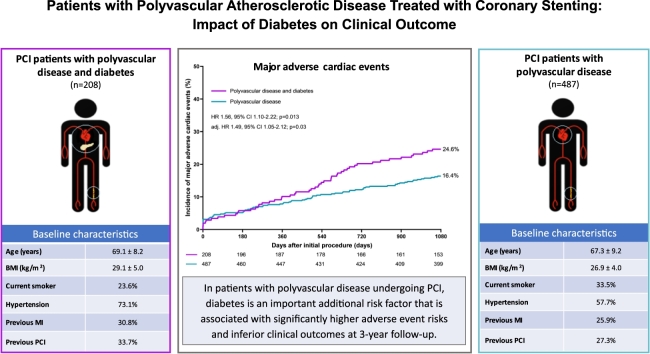

## 1 Introduction

Atherosclerosis is a systemic disease, and the presence of clinically apparent atherosclerosis in one vascular bed is associated with an increased likelihood of symptomatic arterial changes in other vascular beds.[1] Consequently, patients with obstructive coronary artery disease (CAD) are more likely to have concomitant peripheral artery disease, cerebrovascular disease, and aortic disease.[2] These patients with polyvascular disease are at an elevated risk for various adverse events.[3,4] In all-comer patients with obstructive CAD who undergo percutaneous coronary intervention (PCI), the presence of polyvascular disease has been observed in at least 5 % of the cases.[[5], [6], [7]] As previously demonstrated, patients with polyvascular disease are at a markedly elevated risk of adverse clinical events following PCI with contemporary drug-eluting stents (DES), including an almost twice as high mortality as compared to patients without polyvascular disease at long-term follow-up.[5,8,9].

Diabetes mellitus affects both the function and structure of blood vessels throughout the entire human body. It is a well-established risk factor for atherosclerosis and associated with an increased risk of adverse clinical events following PCI.[[10], [11], [12]] The prevalence of diabetes is increased in the presence of polyvascular disease [5], and in all-diabetic populations (not specifically PCI patients) MACE occurred more frequently in the presence of polyvascular disease.[6,7] In a selected population of ACS patients with polyvascular disease, a higher rate of MACE was observed in the presence of diabetes.[8] Yet, the present study is the first to assess in an all-comer PCI population with polyvascular disease the impact of diabetes on post-PCI clinical outcome.

As the precise extent to which diabetes contributes to the elevated risk of adverse clinical events following PCI in patients with polyvascular disease has yet to be determined, this analysis sought to evaluate the impact of diabetes on long-term clinical outcomes following PCI in these patients, which may affect prognosis and secondary prevention. In other words, the present study addresses the question whether diabetes is still a significant additional risk factor in PCI patients who already have a very high adverse event risk due to the presence of polyvascular disease. Data were used from a pooled patient-level database comprising four randomized PCI trials that assessed new-generation DES in all-comers. In particular, we compared the baseline characteristics and 3-year clinical outcomes after PCI in polyvascular disease patients with and without medically treated diabetes.

## 2 Methods

### 2.1 Study design

In order to conduct this analysis, patient-level data from 4 randomized controlled trials consisting of 9,204 all-comer patients, were pooled: TWENTE (*TWENTE I, NCT01066650*), DUTCH PEERS (TWENTE II, *NCT01331707*), BIO-RESORT (TWENTE III, *NCT01674803*), and BIONYX (TWENTE IV, *NCT02508714*)). The details of the original trials have been previously reported.[[16], [17], [18], [19]] Briefly, the trials were investigator-initiated, assessor- and patient-blinded randomized studies. Patients were eligible for enrolment if they were at least 18 years of age, were able to provide informed consent, and required PCI with the implantation of new-generation DES for the treatment of obstructive CAD. Further study details regarding the 4 original trials can be found in Supplementary Table 1. The TWENTE II-IV trials included patients who presented with any type of coronary syndrome, while the TWENTE I trial enrolled all patients except for those presenting with an acute ST-segment elevation myocardial infarction (STEMI). Overall, patients were enrolled at 9 study sites: 6 Dutch centers, 2 Belgian centers, and 1 Israeli center. In the 3-arm BIO-RESORT trial, randomization between stent types was performed in a 1:1:1 fashion, while in the TWENTE, DUTCH PEERS, and BIONYX trials the randomization was performed in a 1:1 fashion. A custom-designed computer program with random block sizes of 4 and 8 was used for web-based randomizations. Stratification was performed for diabetes in the BIO-RESORT and BIONYX trials, and for sex in the TWENTE and BIONYX trials. The trials complied with the Declaration of Helsinki and the Medical Ethics Committee Twente, and the Institutional Review Boards of all participating centers provided approval for all original trials. All trial participants provided written informed consent. Besides the exclusion criteria as described in the 4 randomized trials, no additional exclusion criteria were made for the current analysis.

**Table 1:** Baseline characteristics of patients, target lesions, and PCI procedures.

	**Diabetes**		**p-value**
	Yes (n = 208)	No (n = 487)	
**General patient characteristics**			
Age (years)	69.1 ± 8.2	67.3 ± 9.2	*0.01*
Female sex	64(30.8)	129(26.5)	0.25
BMI (kg/m^2^)	29.1 ± 5.0	26.9 ± 4.0	*<0.001*
Current smoker	48/208(23.6)	162/485(33.5)	*0.01*
**Medical history**			
Renal failure^a^	31(14.9)	50(10.3)	0.08
Hypertension	152(73.1)	281(57.7)	*<0.001*
Hypercholesterolemia	124/204(60.8)	263/479(54.9)	0.16
Previous stroke	33(15.9)	57(11.7)	0.14
LVEF < 30 %	10(4.9)	22(4.6)	0.87
Previous myocardial infarction	64(30.8)	126(25.9)	0.19
Previous PCI	70(33.7)	133(27.3)	0.09
Previous coronary artery bypass grafting	36(17.3)	64(13.1)	0.15
Family history of coronary artery disease	94/201(46.8)	243/469(51.8)	0.23
Coronary syndrome at presentation			0.14
Stable angina pectoris	92(44.2)	202(41.5)	
STEMI	16(7.7)	66(13.6)	
Non-STEMI	56(26.9)	112(23.0)	
Unstable angina pectoris	44(21.2)	107(22.0)	
**Target lesions and PCI procedures**			
Target coronary			
Left main stem	8(3.8)	20(4.1)	0.87
Right coronary artery	93(44.7)	217(44.6)	0.97
Left anterior descending artery	89(42.8)	187(38.4)	0.28
Left circumflex artery	59(28.4)	162(33.3)	0.20
Bypass graft	11(5.3)	23(4.7)	0.75
Total stent length (mm)	42.4 ± 29.7	43.1 ± 30.7	0.39
At least 1 severely calcified lesion	64(30.8)	130(26.7)	0.27
At least 1 ostial lesion	18(8.7)	57(11.7)	0.24
At least 1 bifurcation lesion^b^	65(31.3)	145(29.8)	0.70
At least 1 chronic total occlusion	6(2.9)	24(4.9)	0.23
At least 1 complex lesion^c^	169(81.3)	367(75.4)	0.09
Small vessel treated (<2.75 mm)	135(64.9)	285(58.5)	0.12
Multivessel treatment	46(29.1)	112(23.0)	0.80

Values are mean ± SD, n (%) or n/N (%).

a Defined as previous renal failure, creatinine ≥ 130 μmol/L, or need for dialysis; ^b^Target lesions were classified as bifurcated if a side branch ≥ 1.5 mm originated from them. ^c^Defined as a lesion complexity of B2 or C. Abbreviations: LVEF = Left ventricle ejection fraction; STEMI=ST-segment–elevation myocardial infarction; PCI = percutaneous coronary intervention; BMI = body–mass-index.

The PCI patients (who by definition all had obstructive CAD) were classified as having *polyvascular disease* in the presence of concomitant peripheral arterial disease (PADs). In accordance with the methodology employed in previous studies,[20,21] patients were classified as having *PADs* if they by anamnesis or medical record had a documented history of at least one of the following: (1) symptomatic atherosclerotic lesion in the lower or upper extremities; (2) atherosclerotic lesion in the aorta causing symptoms or requiring treatment; (3) atherosclerotic lesion in the carotid or vertebral arteries related to non-embolic ischemic cerebrovascular event; or (4) symptomatic atherosclerotic lesion in a mesenteric artery.

### 2.2 Procedures, follow-up, and monitoring

All interventional procedures were performed by experienced interventional cardiologists in consonance with current international guidelines. Generally, dual antiplatelet therapy was prescribed for 12 months in case of an acute coronary syndrome, while this was 6 to 12 months for patients with a chronic coronary syndrome. Data on cardiovascular risk factors and comorbidities were collected at the time of the index PCI from the medical record or via questionnaires. Clinical events were assessed annually after the first PCI at a visit to an outpatient clinic, or by questionnaires or telephone follow-up. Adverse clinical events were confirmed by collecting the source documents from hospitals or treating physicians. The original trials were independently monitored. Potential adverse clinical events were adjudicated by external clinical event committees.

### 2.3 Clinical endpoints

The main endpoint of the present analysis was major adverse clinical events (MACE), a composite of any myocardial infarction (MI), emergent coronary bypass surgery, clinically indicated target lesion revascularization, or all-cause mortality. Several other clinical endpoints were assessed, including the composite endpoints target vessel failure (TVF; cardiac mortality, target vessel MI, or clinically indicated target vessel revascularization), and the individual components of these composite clinical endpoints. All clinical endpoints were prespecified according to recommendations of the Academic Research Consortium.[22,23].

### 2.4 Supplementary analyses

We identified trial participants *without* polyvascular disease from the pooled database of the four randomized TWENTE trials and compared the clinical outcomes between those with and without diabetes. Adverse clinical events were assessed in analogy with the main analysis, applying the same endpoint definitions and statistical methods. The results of this analysis are presented in the Supplementary Material and allow contextualization of the findings of the main analysis in the study population *with* polyvascular disease. In addition, we performed another supplementary analysis, assessing the clinical outcomes in patients with polyvascular disease, classified by diabetes and insulin treatment.

### 2.5 Statistical analysis

Differences in categorical variables were assessed with the Chi-square test, and the Student *t*-test or Wilcoxon Rank Sum test were used to assess differences in continuous variables whichever was appropriate. Kaplan-Meier statistics were used to assess time to primary and secondary endpoints, and the log-rank test was applied for between-group comparisons. Cox proportional hazards analysis were used to compute Hazard ratios (HR) with two-sided confidence intervals (CIs). Potential confounders were identified if a p-value of < 0.15 was found in univariate analyses, both in the association of the potential confounder with diabetes and with MACE. In the first pass of a multivariate Cox regression model, all potential confounders were included. Stepwise backward selection was used to exclude variables with a non-significant association with the main endpoint; the final model consisted of only age and sex. Adding potential confounders hypertension, BMI and hypercholesterolemia or randomized stent arm to the final model did not have an impact on the adjusted hazard ratio of the primary endpoint MACE, and were therefore not included in the final model. Confidence intervals were two-sided and p-values < 0.05 were considered significant. SPSS version 24.00 (IBM, Armonk, NY) was used for the statistical analyses.

## 3 Results

### 3.1 Study population

**Fig. 1:**
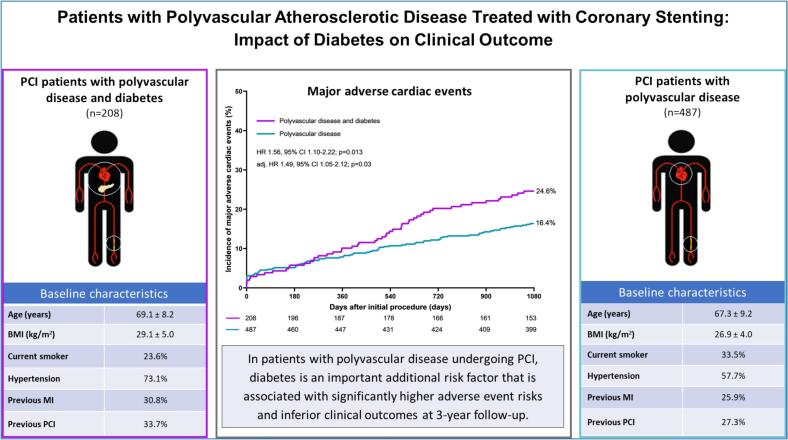
Graphical abstract.

**Fig. 2:**
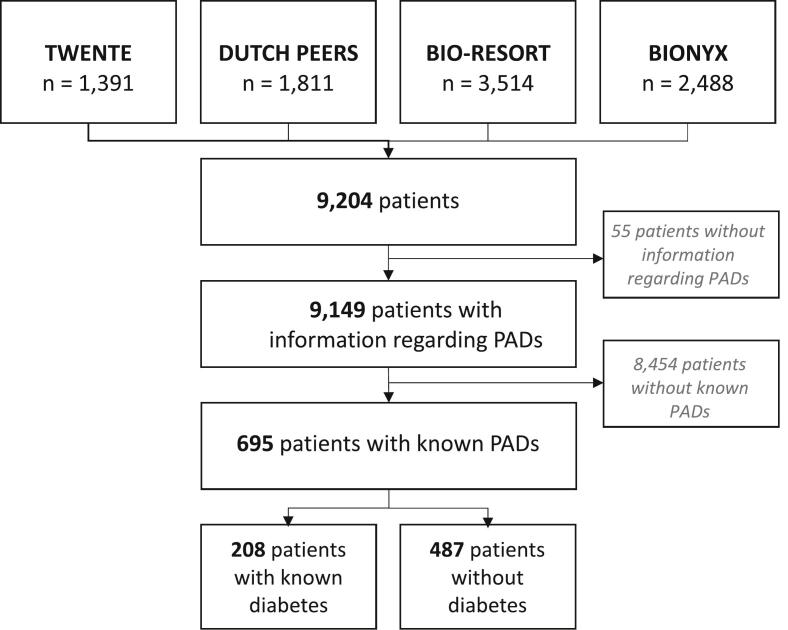
Study flowchart.

The current study population is based on 695 patients with known polyvascular disease. As shown in Fig. 1, Fig. 2, a total of 208 (29.9 %) of the study patients had known, medically treated diabetes while 487 (70.1 %) had no diabetes.

### 3.2 Baseline characteristics

Patients with diabetes were on average 1.8 years older than patients without diabetes, had a higher body-mass-index (BMI) and a higher prevalence of arterial hypertension, but were less likely to be active smokers at the time of the index procedure ***(***Table 1***).*** There was no statistically significant between-group difference in the characteristics of target lesions and PCI procedures. In both patients with and without diabetes, three out of four patients were treated for at least one complex coronary lesion. Further details on the patient, lesion, and procedural characteristics are presented in Table 1.

### 3.3 Clinical outcome

**Fig. 3:**
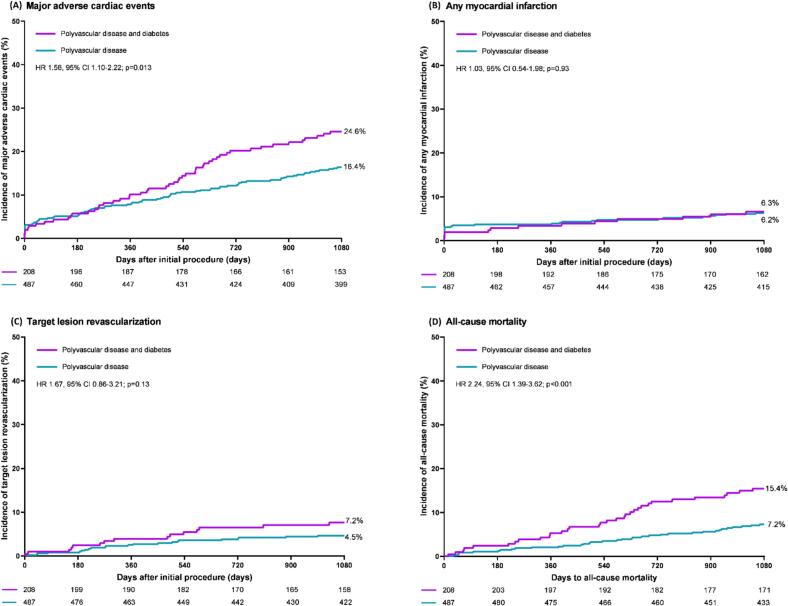
Kaplan-Meier time-to-event curves of the incidence of major adverse cardiac events and individual components: Major adverse cardiac events (A), any myocardial infarction (B), target lesion revascularization (C), and all-cause mortality (D). **Abbreviations:** PADs = peripheral arterial disease, HR = hazard ratio, CI = confidence interval.

At 3-year follow-up, the study population of PCI patients with polyvascular disease showed a significantly higher incidence of the primary endpoint of MACE in the presence of diabetes (24.6 % vs. 16.4 %, HR: 1.56, 95 %CI: 1.10–2.22, p = 0.01). The difference in MACE was primarily attributable to a disparity in its component all-cause mortality (15.4 % vs. 7.2 %, HR: 2.24, 95 %CI: 1.39–3.62, p < 0.001), and it remained statistically significant following adjustment for confounders (adj. HR: 1.49, 95 %CI: 1.05–2.12, p = 0.03). The event rates of the two other individual components of MACE – target lesion revascularization and any MI – showed no statistically significant difference between patients with and without diabetes (7.2 % vs. 4.5 %, p = 0.13, and 6.3 % vs. 6.2 %, p = 0.93, respectively). The time-to-event curves of MACE and its three individual components are displayed in Fig. 3.

The secondary composite endpoint of TVF also showed a higher event rate in patients with diabetes as compared to those without diabetes (20.7 % vs. 14.0 %, HR: 1.53, 95 %CI: 1.04–2.24, p = 0.03). Of the individual components of TVF, both target vessel revascularization and cardiac mortality showed higher rates in patients with diabetes (12.0 % vs. 7.0 %, p = 0.021, and 7.2 % vs. 3.5 %, p = 0.03, respectively), while the there was no difference in target vessel MI (4.8 % vs. 5.5, p = 0.72). The between-group difference in TVF remained statistically significant after adjustment for confounders (adj. HR: 1.48 95 %CI: 1.01–2.18, p = 0.04). Further details on these and other clinical outcomes are presented in Table 2.

**Table 2:** Clinical outcomes at 3-year follow-up.

	**Diabetes**		**HR** **(95 %-CI)**	**P_log-rank_**	**Adjusted HR^c^** **(95 %-CI)**	**P-value**
	Yes (n = 208)	No (n = 487)				
Major adverse cardiac events ^a^	51(24.6)	79(16.4)	1.56 (1.10–2.22)	*0.01*	1.49 (1.05–2.12)	*0.03*
All-cause mortality	32(15.4)	35(7.2)	2.24 (1.39–3.62)	*<0.001*	2.07 (1.28–3.35)	*0.003*
Cardiac mortality	15(7.2)	17(3.5)	2.15 (1.08–4.31)	*0.03*	1.99 (1.00–3.99)	0.05
Any myocardial infarction	13(6.3)	30(6.2)	1.03 (0.54–1.98)	0.93	1.02 (0.53–1.95)	0.96
Target vessel related myocardial infarction	10(4.8)	27(5.5)	0.88 (0.42–1.81)	0.72	0.86 (0.42–1.79)	0.69
Any revascularization	28(13.5)	59(12.1)	1.15 (0.73–1.80)	0.55	1.16 (0.74–1.82)	0.53
Target vessel revascularization	25(12.0)	34(7.0)	1.82 (1.09–3.05)	*0.02*	1.88 (1.12–3.16)	*0.02*
Target lesion revascularization	15(7.2)	22(4.5)	1.67 (0.86–3.21)	0.13	1.72 (0.89–3.33)	0.11
Target vessel failure^b^	43(20.7)	68(14.0)	1.53 (1.04–2.24)	*0.03*	1.48 (1.01–2.18)	*0.04*
Definite-or-probable stent thrombosis	3(1.4)	5(1.0)	1.44 (0.34–6.03)	0.62	1.50 (0.36–6.35)	0.58

Data are n (%), unless otherwise indicated. ^a^Major adverse cardiac events is a composite of all-cause mortality, any myocardial infarction, emergent coronary artery bypass surgery, and clinically indicated target lesion revascularization. ^b^Target vessel failure is a composite of cardiac mortality, target vessel related myocardial infarction, and clinically indicated target vessel revascularization. ^c^Adjusted for sex and age.

### 3.4 Supplementary analysis

For the purpose of comparison with the clinical outcomes of the present study population, Supplementary Table 2 presents the clinical outcomes of 8,454 PCI trial participants patients *without* polyvascular disease. A total of 1,539 (18.2 %) of them had known diabetes and showed significantly worse 3-year outcomes for all clinical endpoints: the rate of MACE was 16.7 % in the presence of diabetes and 9.5 % in the absence of diabetes (HR: 1.83, 95 %CI 1.6–2.1, p < 0.001). After adjustment for confounders, the between-group differences with higher event rates in the presence of diabetes remained statistically significant for MACE (adj. HR: 1.70, 95 %CI: 1.47–1.96, p < 0.001) and all other clinical endpoints. Supplementary Table 3 reports the findings of a subanalysis to assess the clinical outcomes among polyvascular disease patients with insulin-treated diabetes versus no diabetes (Table S3a), and between those with non-insulin-treated diabetes versus no diabetes (Table S3b). In total, 83 (39.9 %) patients with diabetes were treated with insulin. The insulin-treated patients showed, also after adjustment for confounders, a statistically higher rate of MACE than patients without diabetes (31.3 % vs. 16.4 %, HR: 2.00 95 % CI: 1.29–3.12, p = 0.002). In contrast, there was no significant difference in MACE between non-insulin-treated patients and those without diabetes (20.0 % vs. 16.4 %, HR: 1.27 95 %CI: 0.81–1.99, p = 0.30). All-cause mortality showed significantly higher rates in both insulin-treated and non-insulin-treated diabetic patients as compared to patients without diabetes, but the target lesion revascularization rate showed a significant difference only between insulin-treated and non-diabetic patients (9.6 % vs. 4.5 %, p = 0.047).

## 4 Discussion

### 4.1 Study findings

The current analysis reports the additional impact of diabetes in PCI patients with polyvascular disease, which was primarily driven by patients who were insulin-treated. At long-term follow-up, patients with diabetes showed a 56 % higher incidence of the primary endpoint than patients without diabetes (MACE: 24.6 % versus 16.4 %), a difference that was primarily attributable to its individual component all-cause mortality. In addition, several secondary endpoints (TVF and repeat target vessel revascularization) were significantly increased in the presence of diabetes. The absolute increase in MACE rate associated with the presence of diabetes in the present study population was similar to that in patients without polyvascular disease (+8.2 % and + 7.2 %, respectively). The findings of this study indicate that in the high-risk population of PCI patients *with* polyvascular disease, the presence of diabetes represents a profoundly relevant additional cardiovascular risk factor that is associated with inferior clinical outcomes at long-term follow-up after coronary stenting.

### 4.2 Cardiovascular risk profile

Given that atherosclerosis is a systemic disease, patients with obstructive CAD frequently have atherosclerosis in other vascular beds. At least 5 % of all PCI patients have polyvascular disease, meaning they not only have CAD but also PADs.[[5], [6], [7]] Previous studies on clinical outcome after PCI have shown that polyvascular disease is an independent predictor of adverse clinical outcome.[5,6] An analysis of the e-Ultimaster study has shown higher event rates of the composite primary endpoint TLF after PCI in patients with multisite arterial disease than in those without.[5] Another clinical trial has shown that almost half of all patients hospitalized with acute myocardial infarction had known vascular disease.[24] As the number of affected vascular sites increased, the mean age of patients was higher, and patients more often had a history of dyslipidaemia, heart failure, and diabetes. In addition, the odds of major adverse cardiovascular and cerebrovascular events, mortality, and major bleeding incrementally increased and were highest in those with at least three affected vascular beds.[24] Furthermore, a post-hoc analysis of a pharmacological trial has examined the effect of an increasing number of affected vascular beds on the risks of MACE and all-cause mortality in patients with diabetes.[25] At baseline, patients with an increasing number of affected arterial beds were associated with a higher rate of hypertension, dyslipidemia and renal insufficiency. After 5 years of follow-up, each additional diseased bed was associated with higher risks of MACE and all-cause mortality.

In the present study, polyvascular disease patients with diabetes had a higher cardiovascular risk profile with significantly higher age and BMI, and a higher rate of hypertension than those without diabetes. In addition, the prevalence of other comorbidities such as renal insufficiency, dyslipidemia, previous stroke, heart failure, and previous revascularization was numerically higher, which is consistent with the findings of previous studies.[11,12,26].

### 4.3 Outcome of PCI patients with polyvascular disease and diabetes

Although several studies assessed the prevalence and impact of polyvascular disease in an all-diabetic patient population [13,14], very few studies have evaluated the impact of diabetes on clinical outcome in patients with polyvascular disease,[27] especially in patients undergoing PCI.[15] The IMPROVE-IT trial investigated the long-term cardiovascular risk associated with polyvascular disease (defined as peripheral artery disease or previous stroke or transient ischaemic attack) and diabetes in patients with an acute coronary syndrome.[15] After 7 years of follow-up, the presence of diabetes in addition to polyvascular disease was independently associated with an increased risk of reaching the primary composite endpoint of stroke, major coronary event, or cardiovascular death as compared to patients without diabetes (60.0 % vs. 39.8 %). Assessing a slightly different primary composite clinical endpoint at 3-year follow-up after PCI with new-generation DES, the present study reaches a similar conclusion. Nevertheless, in the IMPROVE-IT trial no more than 82 % of the patients with polyvascular disease underwent a PCI, and the study enrolled patients from 2005 to 2010, which implies that in IMPROVE-IT many PCI procedures have been performed with stents of an earlier generation than used in the present study. To our knowledge, this is the first study to assess in an all-comer PCI population with polyvascular disease the impact of diabetes on post-PCI clinical outcome.

Findings of the present study showed a significantly higher rate of MACE in PCI patients with both polyvascular disease and diabetes. Yet, this higher event rate is driven by all-cause mortality and not by differences in any MI or any revascularization. We can only hypothesize about causal mechanisms. At first, patients with diabetes are at higher risk of having autonomic neuropathy, possibly leading to silent myocardial ischemia and consequently, underdiagnosing of myocardial infarction.[28] In addition, it is possible that the incremental effect of diabetes is less noticeable, as both polyvascular disease and diabetes are independent risk factors of cardiac events post-PCI. Finally, we observed that patients with diabetes are at higher risk of all-cause mortality, making it possible that patients died of non-cardiac causes, before experiencing a cardiac event (competing risk phenomenon).

### 4.4 Clinical implications

As the presence of diabetes further complicates the prognosis after PCI in patients with polyvascular disease, secondary prevention of diabetes is of paramount importance. This is especially true for patients with diabetes treated with insulin, who show higher rates of MACE, all-cause mortality and target lesion revascularization. Given the increasing prevalence of diabetes and its strong association with PADs, the current ESC guidelines on the management of peripheral arterial and aortic diseases recommend screening for (pre)diabetes in patients with PADs and, if present, additional medical management to reduce the risk of adverse events.[29] Although this recommendation is based on several studies that have shown a beneficial effect of adding GLP-1 receptor agonists or SGTL2 inhibitors to the standard medical treatment in patients with both diabetes and PADs, it is still unclear whether this therapy should be further intensified in patients with polyvascular disease to reduce the additional risk of cardiovascular events.[[30], [31]].

### 4.5 Limitations

The four original randomized clinical trials were conducted with great care, including external monitoring and independent clinical event adjudication. Nevertheless, the present study has several limitations. Given the post-hoc nature of the analysis, the results should be regarded as hypothesis-generating. As is the case with all clinical studies, the presence of undetected or unmeasured confounders cannot be excluded. As a consequence of the definition of PADs used, undiagnosed and subclinical PADs were not considered. Similar to many other clinical studies, data on risk factors and comorbidities were collected from medical records and questionnaires at the time of enrollment in the randomized trials, while data on risk factors that might have developed during the period of follow-up were not available. Data on the number of affected vascular beds were not available in our database and could therefore not be assessed. In addition, detailed information on anti-diabetic therapy, besides insulin treatment, were not available.

## 5 Conclusion

In the high-risk population of PCI patients with polyvascular disease, the presence of diabetes represents a profoundly significant additional risk factor at long-term follow-up, associated with significantly higher adverse event risks – most evident in insulin-treated patients. Still, MACE was found to be primarily driven by all-cause mortality rather than device-related events or complications following PCI.

Ethics approval and consent to participate

The trials complied with the Declaration of Helsinki and were approved by the Medical Ethics Committee Twente (TWENTE reference number P08-18, DUTCH PEERS reference number P10-29, BIO-RESORT reference number P12-22; BIONYX reference number P15-19) and the Institutional Review Boards of all centers. All patients provided written informed consent.

Financial support

The present study was performed without any external funding. The department Cardiovascular Research and Education of Medisch Spectrum Twente has received institutional research grants provided by Abbott Vascular, Biotronik, Boston Scientific, and Medtronic, not related to the research question of the present study.

Artificial intelligence use

In the process of composing this manuscript, the authors employed the complimentary version of an artificial intelligence-based translation tool (DeepL; https://deepl.com) to evaluate some sentences, enhance the language level, and find alternative expressions. While this did enhance the readability of the manuscript, it did not affect the content.

## CRediT authorship contribution statement

**Daphne van Vliet:** Writing – review & editing, Supervision, Investigation. **Tineke H. Pinxterhuis:** Writing – original draft, Visualization, Validation, Project administration, Methodology, Formal analysis. **Eline H. Ploumen:** Writing – review & editing, Visualization, Validation, Project administration, Methodology. **Marlies M. Kok:** Writing – review & editing, Supervision, Investigation. **Rosaly A. Buiten:** Writing – review & editing, Supervision, Investigation. **Paolo Zocca:** Writing – review & editing, Supervision, Investigation. **Ariel Roguin:** Writing – review & editing, Supervision, Investigation. **Carl E. Schotborgh:** Writing – review & editing, Supervision, Investigation. **Rutger L. Anthonio:** Writing – review & editing, Supervision, Investigation. **Peter W. Danse:** Writing – review & editing, Supervision, Investigation. **Edouard Benit:** Writing – review & editing, Supervision, Investigation. **Adel Aminian:** Writing – review & editing, Supervision, Investigation. **Carine J.M. Doggen:** Writing – review & editing, Supervision, Methodology, Conceptualization. **Clemens von Birgelen:** Writing – review & editing, Supervision, Investigation.

## Declaration of competing interest

The authors declare that they have no known competing financial interests or personal relationships that could have appeared to influence the work reported in this paper.
