# Supplementary material for: Impact of diabetes on three-year outcome after coronary stenting in patients with polyvascular atherosclerotic disease – a secondary analysis of the randomized TWENTE trials
Source: Int J Cardiol Heart Vasc. 2025 Jul 6;59:101741. doi: 10.1016/j.ijcha.2025.101741 (PMC12272603; doi:10.1016/j.ijcha.2025.101741)
Supplement: Supplementary Data 1 [file mmc1.docx]

**Supplemental material**

**Patients with Polyvascular Atherosclerotic Disease Treated with Coronary Stenting: Impact of Diabetes on Clinical Outcome**

Daphne van Vliet, MD^a,b^, Tineke H. Pinxterhuis MD^a,b^, Eline H. Ploumen MD PhD^a,b^,

Marlies M, Kok, MD PhD^a^, Ariel Roguin, MD PhD^c^, Paolo Zocca, MD PhD^a^,

Carl E. Schotborgh MD^d^, Rutger L. Anthonio MD PhD^e^, Peter W. Danse MD PhD^f^,

Edouard Benit MD^g^, Adel Aminian MD^h^, Carine J. M. Doggen PhD^b^,

Clemens von Birgelen MD PhD^a,b^

**Table S1. Details of the TWENTE trials**

| Study name | All patients  (n=9,204) | Patients with polyvascular disease | Randomized  drug-eluting stents |
| --- | --- | --- | --- |
| TWENTE I | 1,391 | 102 | Resolute vs. Xience V |
| TWENTE II  DUTCH PEERS | 1,811 | 153 | Resolute Integrity vs. Promus Element |
| TWENTE III  BIO-RESORT | 3,514 | 240 | Synergy/Orsiro vs. Resolute Integrity |
| TWENTE IV  BIONYX | 2,488 | 200 | Resolute Onyx vs. Orsiro |

Medtronic, Santa Rosa, CA. Abbott Vascular, Santa Clara, CA. Boston Scientific, Marlborough, MA. Biotronik, Bülach, Switzerland.

**Table S2. Clinical outcomes at 3-year follow-up in patients *without* polyvascular disease (8,454)**

|  | **Diabetes** | | **HR**  **(95%-CI)** | **P_log-rank_** | **^c^Adjusted HR**  **(95%-CI)** | **P-value** |
| --- | --- | --- | --- | --- | --- | --- |
|  | Yes (n=1,539) | No  (n=6,915) |  |  |  |  |
| Major adverse cardiac events ^a^ | 257 (16.7) | 657 (9.5) | 1.83 (1.58-2.11) | <0.001 | 1.59 (1.39-1.81) | <0.001 |
| All-cause mortality | 118 (7.7) | 257 (3.7) | 2.12 (1.70-2.63) | <0.001 | 1.76 (1.44-2.14) | <0.001 |
| Cardiac mortality | 57 (3.7) | 113 (1.6) | 2.32 (1.69-3.19) | <0.001 | 1.83 (1.37- 2.44) | <0.001 |
| Any myocardial infarction | 93 (6.0) | 243 (3.5) | 1.77 (1.39-2.24) | <0.001 | 1.55 (1.24-1.95) | <0.001 |
| Target vessel related myocardial infarction | 73 (4.7) | 200 (2.9) | 1.67 (1.28-2.19) | <0.001 | 1.44 (1.12-1.86) | 0.005 |
| Any revascularization | 222 (14.4) | 623 (9.0) | 1.68 (1.44-1.96) | <0.001 | 1.61 (1.39-1.86) | <0.001 |
| Target vessel revascularization | 125 (8.1) | 350 (5.1) | 1.67 (1.36-2.04) | <0.001 | 1.69 (1.39-2.04) | <0.001 |
| Target lesion revascularization | 87 (5.7) | 244 (3.5) | 1.65 (1.29-2.11) | <0.001 | 1.59 (1.26-2.01) | <0.001 |
| Target vessel failure^b^ | 217 (14.1) | 566 (8.2) | 1.79 (1.53-2.09) | <0.001 | 1.62 (1.40-1.88) | <0.001 |
| Definite-or-probable stent thrombosis | 29 (1.9) | 61 (0.9) | 2.18 (1.40-3.39) | 0.001 | 1.91 (1.24-2.93) | 0.003 |

Data are n (%), unless otherwise indicated. ^a^Major adverse cardiac events is a composite of all-cause mortality, any myocardial infarction, emergent coronary artery bypass surgery, and clinically indicated target lesion revascularization.  ^b^Target vessel failure is a composite of cardiac mortality, target vessel related myocardial infarction, and clinically indicated target vessel revascularization. ^c^Adjusted HR: age, sex, renal insufficiency, previous MI, at least 1 severely calcified lesion, and previous stroke

**Table S3. Clinical outcomes at 3-year follow-up in patients *with* polyvascular disease, classified by diabetes and insulin treatment**

| **a.** *Follow-up in patients with insulin-treated diabetes versus no diabetes* | | | | | | |
| --- | --- | --- | --- | --- | --- | --- |
|  | **Diabetes** | | **HR**  **(95%-CI)** | **P_log-rank_** | **Adjusted HR^b^**  **(95%-CI)** | **P-value** |
|  | Yes, insulin-treated (n=83) | No diabetes  (n=487) |  |  |  |  |
| Major adverse cardiac events ^a^ | 26(31.3) | 79(16.4) | 2.00 (1.29-3.12) | 0.002 | 1.91 (1.22-2.98) | 0.005 |
| All-cause mortality | 14(16.9) | 35(7.2) | 2.41 (1.30-4.49) | 0.004 | 2.16 (1.16-4.02) | 0.02 |
| Any myocardial infarction | 9(10.8) | 30(6.2) | 1.80 (0.85-3.78) | 0.12 | 1.77 (0.84-3.74) | 0.14 |
| Target lesion revascularization | 8(9.6) | 22(4.5) | 2.19 (0.97-4.91) | 0.05 | 2.29 (1.01-5.19) | 0.047 |
| **b.** *Follow-up in patients with diabetes but no insulin treatment versus no diabetes* | | | | | | |
|  | **Diabetes** | | **HR**  **(95%-CI)** | **P_log-rank_** | **Adjusted HR^b^**  **(95%-CI)** | **P-value** |
|  | Yes, non-insulin-treated (n=125) | No diabetes  (n=487) |  |  |  |  |
| Major adverse cardiac events ^a^ | 25(20.0) | 79(16.4) | 1.27 (0.81-1.99) | 0.30 | 1.22 (0.78-1.91) | 0.39 |
| All-cause mortality | 18(14.4) | 35(7.2) | 2.12 (1.20-3.74) | 0.008 | 2.05 (1.16-3.62) | 0.01 |
| Any myocardial infarction | 4(3.2) | 30(6.2) | 0.53 (0.19-1.49) | 0.22 | 0.52 (0.97-1.05) | 0.21 |
| Target lesion revascularization | 7(5.6) | 22(4.5) | 1.31 (0.56-3.06) | 0.54 | 1.38 (0.59-3.26) | 0.46 |

Data are n (%), unless otherwise indicated. ^a^Major adverse cardiac events is a composite of all-cause mortality, any myocardial infarction, emergent coronary artery bypass surgery, and clinically indicated target lesion revascularization.  ^b^Adjusted HR, based on age and sex.
